# Recreational target games improve sensorimotor performance in boys aged 10–14 years: a randomized controlled trial with 4-week follow-up

**DOI:** 10.3389/fcogn.2026.1968283

**Published:** 2026-10-23

**Authors:** Mehmet Altin

**Affiliations:** Faculty of Sports Sciences, Selçuk University, Konya, Türkiye

**Keywords:** balance, change-of-direction speed, reaction time, sensorimotor performance, target games

## Abstract

This study examined the acute and lasting effects of a recreational target games program on sensorimotor performance in boys aged 10–14 years. Sixty-nine students from Konya, Türkiye were assigned to a Target Games Activity Group (TGAG, *n* = 22), a Control Group (CG, *n* = 24), and an Active Control Group (ACG, *n* = 23) completing a general recreational games program without target-based content. Following orientation and baseline testing, the TGAG completed a 6-week target games program (three sessions per week, 45 min per session); post-test and 4-week follow-up assessments followed. Reaction time, upper-limb movement speed, dynamic balance, and change-of-direction speed were assessed using the Nelson Hand Reaction Test, the Modified Bass Test, the Pro-Agility Test, and the Eurofit Plate Tapping Test. Data were analyzed using Two-Way Repeated Measures ANOVA with Bonferroni *post-hoc* comparisons, supplemented by pre-test-adjusted ANCOVA models testing between-group differences directly. The Time × Group interaction was significant for all four outcomes. Pre-test-adjusted analyses showed the TGAG differed significantly from both control groups at follow-up on all four measures, with effect sizes ranging from small (reaction time, upper-limb movement speed) to large (dynamic balance, change-of-direction speed); its advantage over the ACG was significant at post-test on three measures, emerging only at follow-up for upper-limb movement speed. The ACG's post-test gains, where present, were not retained at follow-up. These findings indicate that recreational target games are a promising intervention for improving sensorimotor performance in boys aged 10–14 years, with gains evident at the 4-week follow-up.

## Introduction

The period between 10 and 14 years of age represents a particularly sensitive developmental window, coinciding with the onset of adolescence and the associated maturation of the frontal lobe, a process closely linked to attentional control and the optimization of reaction time (Blakemore and Choudhury, 2006; Posner and Rothbart, 2007). Reaction speed and speed-related skills have been reported to develop most intensively during this same window (Dovalil, 2002), with reaction time shortening progressively as motor, cognitive, and neurological development proceed in parallel (Vaeyens et al., 2008). This convergence of neurodevelopmental readiness and trainability positions early adolescence as a critical period for interventions targeting sensorimotor performance.

Sporting activity is conventionally framed as a driver of physical development, yet it simultaneously engages a dense network of sensory and cognitive processes, perception, attention, timing, kinesthetic and proprioceptive sensitivity, and spatial awareness among them, that are exercised and refined through movement (Craighero, 2022). Eye–hand coordination in particular is considered a foundational skill that continues to develop through childhood and adolescence, with direct relevance to functional and sport-specific performance (Oberer et al., 2018). This developmental potential is, however, set against a well-documented decline in physical activity across childhood and adolescence (Dumith et al., 2011; Arundell et al., 2013), a decline that appears to sharpen specifically at the primary-to-middle-school transition (Jago et al., 2012; Rutten et al., 2014). Multidimensional exercise programs designed to preserve sensorimotor and visual-motor competence during this vulnerable window are therefore of considerable practical importance.

Play-based and game-based formats offer one promising vehicle for such programs. Structured play has been associated with gains in fine and gross motor skills, coordination, and social interaction in early childhood (Lobo and Winsler, 2006), and is theorized to support psychomotor development by embedding motor responses within an actively explored, self-directed environment (Piaget, 1952; Higgins and Lutz, 2019). Within this broader category, target-oriented games—activities organized around aiming, timing, and precise object manipulation—appear especially well suited to sensorimotor training. In school-aged children, visual-motor coordination and executive functions have been shown to develop together and to jointly support motor performance (Oberer et al., 2018), and structured, repetitive practice has been associated with gains in sport-specific motor skills, including through implicit learning mechanisms that appear particularly effective in primary-school-aged children (van Abswoude et al., 2021). Evidence from applied target-game settings converges on similar benefits: target shooting practice has been shown to improve attention-related cognitive functions—including focus, information processing, and the ability to screen out distracting stimuli—in school-aged children and adolescents in controlled intervention studies (Løhre et al., 2022). Coordination training more broadly has been shown to produce greater gains in technical and coordinative ability than traditional training methods in youth athletes (Ljach and Witkowski, 2010), suggesting that structured, game-based motor practice may constitute an efficient tool for sensorimotor development in this age group.

Despite this converging evidence, research directly testing the effect of recreational target games on visual-motor and sensorimotor performance remains scarce. Prospective, controlled experimental work is therefore needed to determine whether a structured target-games intervention produces genuine, trainable improvements in this domain—and, critically, whether any such improvements are retained once the intervention ends. The present study addresses this gap through a randomized controlled, pre-test/post-test/follow-up design, examining the effect of a 6-week recreational target games program on visual-motor and sensorimotor skills in 10- to 14-year-old boys, and evaluating the retention of any resulting gains 4 weeks after the intervention concluded.

## Methods

### Study design

This study employed a randomized controlled experimental design with pre-test, post-test, and follow-up (retention) measurements to examine the effect of a 6-week structured target games program on selected motor and coordinative parameters in boys aged 10–14 years, and to assess the persistence of these effects.

### Participants

The study group consisted of 69 male middle school students aged 10–14 years, enrolled in schools in Konya, who volunteered to participate. *A priori* power analysis was conducted using G^*^Power (*F* tests–ANOVA: repeated measures, within–between interaction; effect size *f* = 0.25, α err prob = 0.05, power = 0.95; 3 groups, 3 measurements; assumed correlation among repeated measures = 0.50; non-sphericity correction ε = 1) prior to data collection to determine the minimum required sample size. This analysis indicated a minimum total sample size of 54 participants (18 per group) was required to detect the specified effect with the stated power. The final analyzed sample (*n* = 69; 22–24 per group) exceeded this requirement. A pool of 75 male middle school students (25 per group) was initially formed for the three groups. To ensure baseline comparability, participants were stratified by age, height, and weight prior to allocation, and within each stratum were then randomly assigned to the three groups using randomizer.org. Sequence generation and group allocation were both performed by the researcher; a separate allocation-concealment mechanism involving an independent party was not employed, which we acknowledge as a methodological limitation. Male participants were selected because sex-based differences are pronounced among children in the 10–14 age range; the absence of female participants constitutes a limitation of this study. The CONSORT diagram for the study, prepared in accordance with the CONSORT 2025 statement (Hopewell et al., 2025), is presented in Figure 1. Participants' age, height, and weight characteristics are presented in Table 2.

- Target Games Activity Group (TGAG; *n* = 22): the group that received the specially designed recreational target games intervention.
- Active Control Group (ACG; *n* = 23): the group that took part in a general recreational games program without target-based content.
- Control Group (CG; *n* = 24): the group that did not take part in any additional structured exercise or experimental program beyond the standard curriculum.

**Figure 1:**
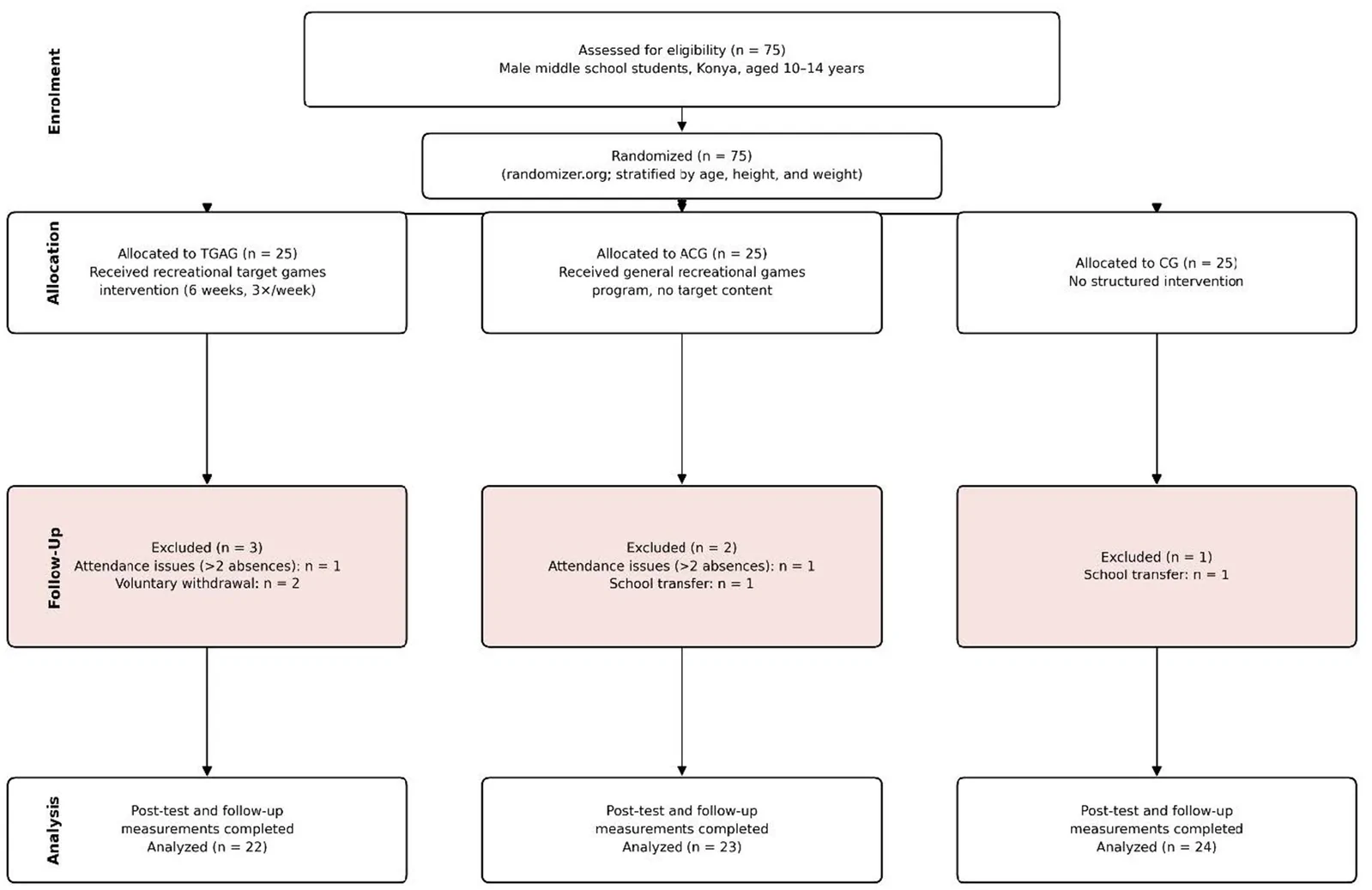
CONSORT flow diagram of participants enrollment, allocation, and analysis.

Although the three groups were initially formed with 25 participants each (*n* = 75), six students were subsequently excluded: three from the TGAG (one due to attendance problems, and two due to voluntary withdrawal), two from the ACG (one due to attendance problems and one due to school transfer), and one from the CG (due to school transfer), leaving a final sample of 69 participants. Analyses were conducted on participants with complete data (per-protocol).

The study was conducted entirely on the grounds and in the gymnasium of a single middle school; this was a deliberate design choice intended to standardize the conditions under which the intervention was implemented.

Prior to the study, all participants and their parents were informed in detail about the purpose, content, and possible risks of the intervention, and an Informed Consent Form was obtained from parents. An orientation period was conducted during Week 1, followed by baseline testing in Week 2, and between-group homogeneity was confirmed through baseline measurements.

## Data collection tools

### Nelson hand reaction test

Used to assess participants' simple hand reaction speed (visual-motor reaction time) under field conditions (Johnson and Nelson, 1986).

- Testing Protocol: the participant was seated upright in a chair, with the forearm and hand resting comfortably on the table, the elbow flexed at 90°, and the hand in a neutral position. The thumb and index finger were held open and parallel, 8–10 cm beyond the edge of the table. The examiner positioned a standard ruler between the participant's thumb and index finger, aligned 10 cm above the hand. The participant was instructed to focus on the ruler and catch it as quickly as possible once released without warning. The distance (d), in centimeters, corresponding to the point where the ruler was caught relative to the upper edge of the thumb, was recorded.
- Scoring and calculation: the test was repeated three times, and the trial with the shortest ruler-drop distance (best performance) was recorded for analysis. The resulting drop distance (d) was converted to meters and transformed into reaction time (t), in seconds, using the following free-fall formula based on gravitational acceleration (g = 9.81 m/s^2^):

Reaction Time (s) = √(2 × Ruler Drop Distance (m)/Gravitational Acceleration (9.81 m/s^2^))

Because only the best of three trials was recorded, this scoring procedure may attenuate measurement variability but could also introduce a selection bias favoring larger apparent improvements if trial-to-trial variability differed between groups or time points; this is addressed further in the Limitations.

Note: this test represents a practical field-based alternative to laboratory light-based reaction devices; it measures only the sudden visual-motor reaction (reflex) response time, not attentional lapses.

### Plate tapping test

The Plate Tapping Test from the Eurofit test battery was administered to assess participants' local segmental movement speed of the upper extremity (arm/hand) (Council of Europe, 1988).

- Testing Protocol: Two plastic discs, 20 cm in diameter with 60 cm between their centers, and a rectangular plate (10 × 20 cm) positioned exactly between them, were fixed to an adjustable table. Standing at the table, the participant placed their non-dominant hand on the central plate and, using their dominant hand, performed consecutive taps between the two discs as rapidly as possible.
- Scoring: the test continued until the hand had completed a total of 25 full cycles (50 taps) between the two discs. The test was administered twice, and the best (shortest) time to complete the 25 cycles was recorded with a stopwatch to a precision of 0.01 s.

### Modified bass dynamic balance test

Administered to measure participants' lower-extremity postural control ability and their capacity to stabilize body balance after a jump (Johnson and Leach, 1968).

- Testing protocol: the test was performed on a course consisting of one starting mark (S) and 10 consecutive footprint markers, positioned on the floor at diagonal angles and fixed distances. The participant jumped on one foot from the starting point to marker 1, stabilized their body balance immediately upon landing, and held the position without disturbance for 5 s. The participant then proceeded in a zigzag pattern, alternating feet, to complete all 10 markers. The 5-s timing was regulated using an audible metronome set to 60 bpm.
- Scoring: the test was scored out of a total of 100 points. At each marker, a Landing Score (maximum 5 points) was awarded for landing on the marker without error, and one point was awarded for each second the position was held without disturbance after landing (maximum 5 points). Points were deducted if the heel touched the ground, the hands left the hips, or the foot slipped on the floor.

### Pro-agility test (20-yard shuttle)

Used to measure participants' pre-planned change-of-direction speed, expressed in seconds (Haff and Triplett, 2016).

- Testing protocol: a 10-yard line was created by placing cones 5 yards (4.57 m) to the right and left of the starting line. An electronic photocell gate was placed at the starting line. Starting from a three-point stance, the participant first sprinted to the right-hand cone (5 yards) and touched the line, then changed direction and sprinted to the left-hand cone (10 yards) and touched it, and finally changed direction again to sprint straight through the starting line.
- Scoring: following one practice trial, the test was administered twice; the time to cross the finish line was measured with an electronic photocell to a precision of 0.01 s, and the best (shortest) time was recorded as change-of-direction score.

### Intervention program

This study was not conducted during Physical Education and Sport class hours, but during the school's Educational Activities class period. While all students continued to take part in the activities specified in the standard curriculum, the TGAG and ACG additionally completed a target games program or a general recreational games program, respectively, during this period. At each session, the start, warm-up, main phase, and closing segments were monitored by the supervising physical education teachers and student assistants, and attendance and session-completion records were kept throughout. Because participants with more than two absences were excluded prior to analysis, all participants included in the final sample attended at least 16 of the 18 scheduled sessions (≥88.9%). No adverse events (e.g., injuries, falls) were reported during either intervention program.

The experimental procedure of the study spanned a total of 12 weeks:

✓ Orientation period (week 1): participants and school administration were briefed, and field adaptation and test familiarization sessions were conducted.
✓ Pre-test/baseline measurements (week 2): baseline levels were established by administering the test battery to all groups (TGAG, ACG, CG).
✓ Experimental intervention phase (weeks 3–8): the TGAG received the “Recreational Target Games Program,” consisting of 18 sessions over 6 weeks, three sessions per week, 45 min per session, of which 5 min were allocated to introduction, organization, and transitions, 5 min to warm-up, 5 min to cool-down, and the remaining time to the main game phase (28–31 min in the TGAG and 27–32 min in the ACG). Within sessions, the TGAG was divided into two subgroups, and each participant was given three attempts per game. To control for general motivational effects, the ACG followed an equivalent session structure and reward scheme while playing simple, non-target-related games. The ACG completed a structurally matched program of general recreational and team-building games (e.g., tug-of-war, tag variants, relay races) that contained no aiming or target-based scoring component, and that was designed to equate the two intervention arms for session duration, team structure, small incentive rewards, and social contact, while isolating the target/aiming component as the variable of interest specific to the TGAG. In both the TGAG and the ACG, winning subgroups were rewarded with small treats (e.g., chocolate) at the end of each session; no ranking-based privilege (such as priority in the following week's attempts) was applied. Both programs were delivered at a recreational, self-selected pace typical of children's play; heart rate, respiratory rate, and rating of perceived exertion were not recorded, and participants were not asked to exert maximal effort at any stage. In Week 7, visual and auditory conditions were progressively altered to introduce sensory constraint: participants wore a blindfold or received color cues through lip-reading with ear protection instead of direct visual or verbal information. This manipulation was grounded in evidence that somatosensory and proprioceptive information contributes to early motor learning (Bernardi et al., 2015; Rosenkranz and Rothwell, 2012) and that restricting visual information can affect performance without necessarily impairing learning (Cates and Gordon, 2024); these sessions were therefore framed as testing the maintenance of throwing accuracy under constrained sensory conditions rather than as a learning manipulation *per se*.
✓ Post-test measurements (end of week 8): the second set of measurements was taken at the end of the intervention program.
✓ Retention/follow-up measurements (Week 12): 4 weeks after the end of the intervention, and without any intervening activity, a third set of measurements was administered to assess the retention of the gains achieved.

The chronological sequence of the 18-session TGAG program is summarized in Table 1; full session-by-session protocols for both the TGAG and the ACG, including game rules, distances, target dimensions, and scoring, are available from the author upon reasonable request.

**Table 1:** The 18-session intervention program administered to the TGAG.

Week/theme	Session	Recreational target game
**Week 3** Fixed targets	Day 1	Hitting bowling pins with a plastic ball from a distance of 4–5 m
	Day 2	Ring toss over the pins
	Day 3	Throwing balls to stick them on a large wheel of fortune
**Week 4** Moving targets	Day 1	Hitting three medicine balls suspended between poles with tennis balls
	Day 2	Rolling a ball through a moving bicycle wheel
	Day 3	Throwing a ball through a hoop held by a moving peer
**Week 5** Accuracy and balance	Day 1	Throwing 10 table-tennis balls into vases while standing on one foot
	Day 2	Repeatedly throwing a plastic ball at a hoop across the room with accuracy
	Day 3	Collecting thrown balls in a basket while moving along a maze path
**Week 6** Time-pressured coordination	Day 1	Passing the maximum number of balls through a target within 30 seconds
	Day 2	Timed shooting while passing through obstacles (team competition)
	Day 3	Using three attempts to shoot at a target while facing away from it, without seeing it
**Week 7** Sensory control and attention	Day 1	Throwing at a hoop with eyes closed, guided by a bell sound
	Day 2	Passing a ball through hoops moving right–left and up–down
	Day 3	Hitting a colored target by lip-reading while wearing headphones
**Week 8** Cognitive and motor control	Day 1	Hitting high numbers on a wheel of fortune without stepping off the line
	Day 2	Throwing a number of balls into a basket equal to the answer of a posed problem
	Day 3	Multi-skill circuit (slalom, obstacle, bench, medicine ball)

**Table 2:** Age, height, and weight characteristics of participants.

Group	*n*	Age (years)		Height (cm)		Weight (kg)	
		M	SD	M	SD	M	SD
TGAG	22	11.91	1.44	149.27	8.42	43.27	6.95
ACG	23	11.87	1.39	147.61	7.33	42.61	5.48
CG	24	11.96	1.33	148.71	7.53	41.88	5.09
Total	69	11.91	1.37	148.52	7.68	42.57	5.81

The TGAG and ACG activity programs, as well as all measurements, were carried out under the coordination of the project lead by two Physical Education and Sport teachers and two undergraduate sport sciences students. All measurements were recorded by the study staff. A separate two-person student team, not otherwise involved in the study, independently cross-checked the manual and electronic records twice to verify the accuracy of data transcription; this procedure confirmed recording accuracy but does not substitute for blinded outcome assessment, as noted in the Limitations.

### Statistical analysis

Data were analyzed using Jamovi (Version 2.3.28). The normality of the data distribution was evaluated using the Shapiro–Wilk and Kolmogorov–Smirnov tests, together with an examination of skewness and kurtosis values.

The homogeneity of variance between groups was examined using Levene's test, and the assumption of homogeneity was found to be met at all measurement time points (*p* > 0.05). In the repeated-measures ANOVA analyses, the assumption of sphericity was tested using Mauchly's test; where this assumption was violated (*p* < 0.05), degrees of freedom were reported using the Greenhouse-Geisser epsilon (ε) correction. As participants were stratified by age, height, and weight prior to randomization, these variables were not included as covariates in the primary analyses, consistent with recommendations for stratified randomized designs.

A Two-Way Repeated Measures ANOVA was used to determine the effects of time, group, and the Time × Group interaction. Bonferroni-corrected *post-hoc* analyses were conducted for pairwise comparisons to identify the source of significant differences and to control Type I error. The level of statistical significance was set at *p* < 0.05.

## Results

Baseline (pre-test) between-group comparisons, tested using one-way ANOVA, revealed no statistically significant differences for any of the four performance variables: Nelson Hand Reaction (*F*_(2, 66)_ = 0.002, *p* = 0.998), Plate Tapping (*F*_(2, 66)_ = 0.010, *p* = 0.990), Modified Bass Dynamic Balance (*F*_(2, 66)_ = 0.081, *p* = 0.923), and Pro-Agility (*F*_(2, 66)_ = 0.117, *p* = 0.890), confirming that the TGAG, ACG, and CG groups were homogeneous at baseline. Levene's test indicated that the assumption of homogeneity of variance was met at all measurement points (all *p* > 0.05). Mauchly's test showed that the assumption of sphericity was violated for all four variables (all *p* < 0.01); accordingly, degrees of freedom were corrected using the Greenhouse-Geisser epsilon. Descriptive statistics by group and time are presented in Table 3, and group-by-time changes for each outcome are illustrated in Figures 2–5. The results of the two-way repeated-measures ANOVA are presented in Table 4.

**Table 3:** Descriptive statistics (M ± SD) by group and measurement time.

Variable	Group	Pre-test	Post-test	Follow-up
Nelson hand reaction test (ms)	TGAG (*n* = 22)	188.13 ± 8.60	184.01 ± 8.88	184.49 ± 8.89
	ACG (*n* = 23)	188.00 ± 7.99	186.18 ± 7.65	187.89 ± 7.77
	CG (*n* = 24)	187.99 ± 6.53	187.58 ± 6.48	187.58 ± 5.98
Plate tapping (s)	TGAG (*n* = 22)	12.51 ± 0.76	12.28 ± 0.74	12.30 ± 0.73
	ACG (*n* = 23)	12.51 ± 0.76	12.36 ± 0.74	12.56 ± 0.79
	CG (*n* = 24)	12.54 ± 0.92	12.55 ± 0.91	12.55 ± 0.90
Modified bass balance (points)	TGAG (*n* = 22)	76.86 ± 2.82	82.82 ± 3.76	81.95 ± 4.33
	ACG (*n* = 23)	76.61 ± 3.07	79.26 ± 2.18	76.39 ± 2.87
	CG (*n* = 24)	76.50 ± 3.35	76.17 ± 3.27	76.29 ± 3.18
Pro-agility (s)	TGAG (*n* = 22)	5.27 ± 0.26	5.02 ± 0.20	5.04 ± 0.20
	ACG (*n* = 23)	5.23 ± 0.33	5.21 ± 0.31	5.30 ± 0.23
	CG (*n* = 24)	5.24 ± 0.27	5.27 ± 0.26	5.24 ± 0.28

TGAG, Target games activity group; ACG, Active control group; CG, control group. Lower values indicate better performance for Nelson hand reaction, Plate tapping, and Pro-agility; higher values indicate better performance for the modified bass dynamic balance test.

**Figure 2:**
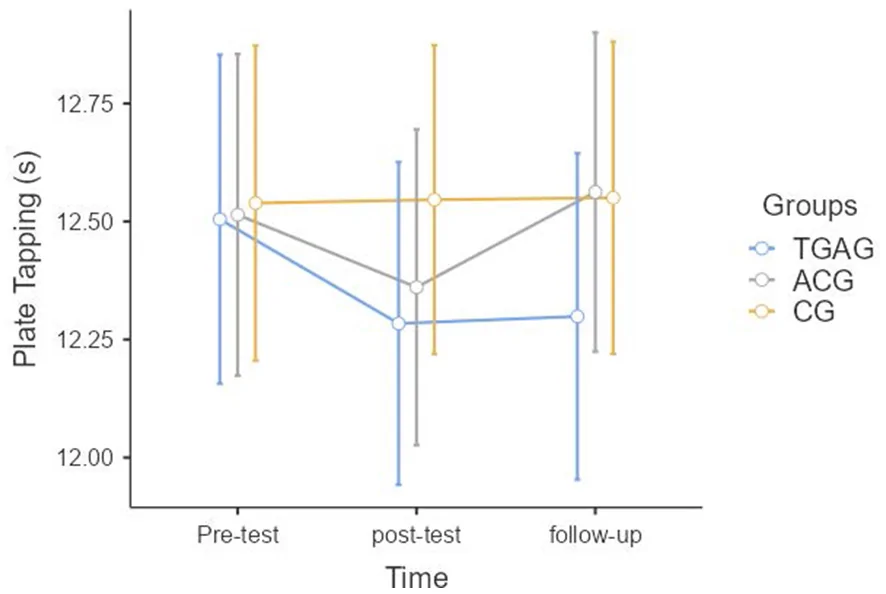
Change in plate tapping test performance by group and measurement time.

**Figure 3:**
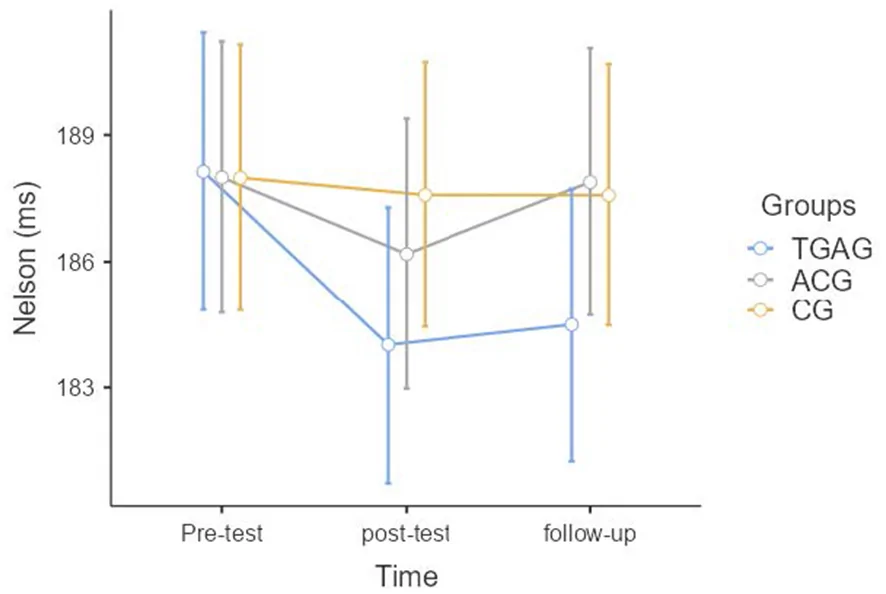
Change in Nelson hand reaction test performance by group and measurement time.

**Figure 4:**
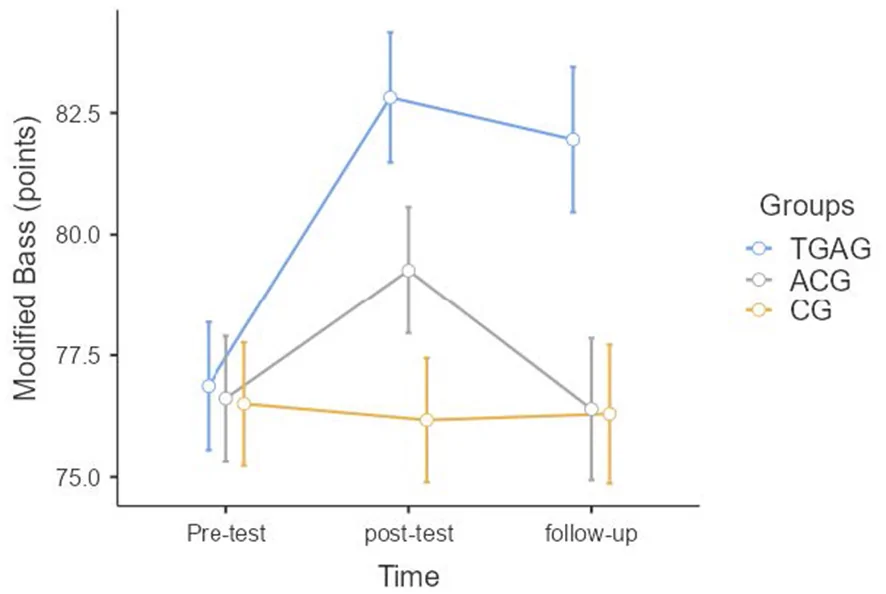
Change in Modified bass balance test performance by group and measurement time.

**Figure 5:**
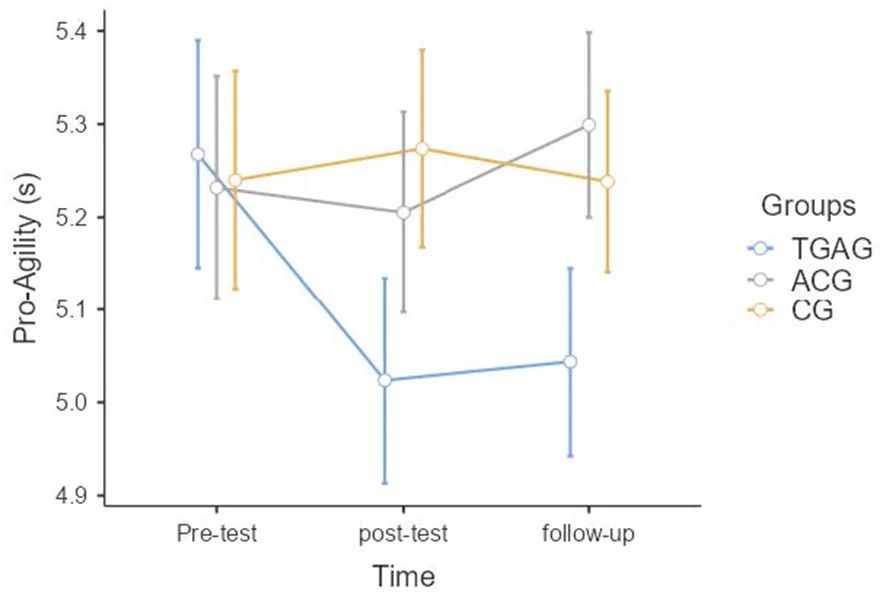
Change in Pro-agility test performance by group and measurement time.

**Table 4:** Two-way repeated-measures ANOVA results for the performance variables.

Variable	Effect	*df*	*F*	*p*	*ηp* ^2^
Nelson hand reaction test (ms)	Time	1.42, 93.77	50.22	< 0.001	0.43
	Group	2, 66	0.53	0.592	0.02
	Time × group	2.84, 93.77	19.41	< 0.001	0.37
Plate tapping test (s)	Time	1.73, 114.48	19.83	< 0.001	0.23
	Group	2, 66	0.30	0.742	0.01
	Time × group	3.47, 114.48	12.95	< 0.001	0.28
Modified bass dynamic balance test (score)	Time	1.75, 115.60	64.17	< 0.001	0.49
	Group	2, 66	11.89	< 0.001	0.26
	Time × group	3.50, 115.60	39.94	< 0.001	0.55
Pro-agility test (s)	Time	1.69, 111.22	12.70	< 0.001	0.16
	Group	2, 66	2.21	0.118	0.06
	Time × group	3.37, 111.22	20.93	< 0.001	0.39

Greenhouse-Geisser corrected values are reported for all within-subject effects, as the sphericity assumption was violated. TGAG, target games activity group; ACG, active control group; CG, control group.

### Nelson hand reaction test

The main effect of time was significant (*F*_(1.42, 93.77)_ = 50.22, *p* < 0.001, η*p*^2^ = 0.43), whereas the main effect of group was not (*F*_(2, 66)_ = 0.53, *p* = 0.592, η*p*^2^ = 0.02). The Time × Group interaction was significant (*F*_(2.84, 93.77)_ = 19.41, *p* < 0.001, η*p*^2^ = 0.37). Bonferroni-adjusted pairwise comparisons (Table 5) showed that the TGAG improved significantly from pre- to post-test (4.12 ms, *p* < 0.001) and from pre-test to follow-up (3.64 ms, *p* < 0.001), with no significant change between post-test and follow-up (−0.48 ms, *p* = 1.000), indicating that the gains were retained. In the ACG, the pre-to-post improvement was significant (1.81 ms, *p* < 0.001), but performance declined significantly from post-test to follow-up (−1.70 ms, *p* < 0.001), and the pre-test–follow-up difference was no longer significant (*p* = 1.000), indicating that the initial gains were lost by follow-up. No significant change was observed in the CG at any time point (all *p* = 1.000).

**Table 5:** Bonferroni-adjusted pairwise comparison results for within-group time effects.

Variable	Group	Comparison	Mean Diff.	*p*	Hedges g
Nelson hand reaction test	TGAG	Pre-post	+4.12	< 0.001^*^	0.46
		Pre-follow-up	+3.64	< 0.001^*^	0.41
		Post-follow-up	−0.48	1.000	−0.05
	ACG	Pre-post	+1.81	< 0.001^*^	0.23
		Pre-follow-up	+0.11	1.000	0.01
		Post-follow-up	−1.70	< 0.001^*^	−0.22
	CG	Pre-post	+0.41	1.000	0.06
		Pre-follow-up	+0.41	1.000	0.06
		Post-follow-up	+0.01	1.000	0.00
Plate tapping test	TGAG	Pre-post	0.22	< 0.001^*^	0.30
		Pre-follow-up	0.21	< 0.001^*^	0.28
		Post-follow-up	−0.01	1.000	−0.03
	ACG	Pre-post	0.15	< 0.001^*^	0.20
		Pre-follow-up	−0.05	1.000	−0.06
		Post-follow-up	−0.20	< 0.001^*^	−0.26
	CG	Pre-post	−0.01	1.000	−0.01
		Pre-follow-up	−0.01	1.000	−0.01
		Post-follow-up	0.00	1.000	0.00
Modified bass dynamic balance test	TGAG	Pre-post	−5.95	< 0.001^*^	−1.76
		Pre-follow-up	−5.09	< 0.001^*^	−1.37
		Post-follow-up	0.86	0.583	0.21
	ACG	Pre-post	−2.65	< 0.001^*^	−0.98
		Pre-follow-up	0.22	1.000	0.07
		Post-follow-up	2.87	< 0.001^*^	1.11
	CG	Pre-post	0.33	1.000	0.10
		Pre-follow-up	0.21	1.000	0.06
		Post-follow-up	−0.13	1.000	−0.04
Pro-agility test	TGAG	Pre-post	0.24	< 0.001^*^	1.06
		Pre-follow-up	0.22	< 0.001^*^	0.97
		Post-follow-up	−0.02	1.000	−0.10
	ACG	Pre-post	0.03	1.000	0.06
		Pre-follow-up	−0.07	1.000	−0.24
		Post-follow-up	−0.09	0.054	−0.32
	CG	Pre-post	−0.03	1.000	−0.11
		Pre-follow-up	0.00	1.000	0.00
		Post-follow-up	0.04	1.000	0.11

TGAG, target games activity group; ACG, active control group; CG, control group. Pre, pre-test, Post, post-test, Follow-up, retention measurement. ^*^p < 0.05 (Bonferroni-adjusted).

To directly test between-group differences in change, pre-test-adjusted ANCOVA models were additionally conducted for the post-test and follow-up scores (Table 6). After adjusting for pre-test values, post-test reaction time differed significantly between groups (*F*_(2, 65)_ = 21.90, *p* < 0.001, η*p*^2^ = 0.40). The TGAG showed a shorter adjusted post-test reaction time than both the ACG (adjusted mean difference = 2.30 ms, *p* < 0.001) and the CG (3.71 ms, *p* < 0.001), and the ACG was also significantly faster than the CG (1.41 ms, *p* = 0.042). At follow-up, the group difference remained significant (*F*_(2, 65)_ = 19.03, *p* < 0.001, η*p*^2^ = 0.37): the TGAG remained significantly faster than both the ACG (3.53 ms, *p* < 0.001) and the CG (3.22 ms, *p* < 0.001), whereas the ACG–CG difference was no longer significant (0.30 ms, *p* = 1.000), confirming that the ACG's initial advantage over the CG had disappeared by follow-up.

**Table 6:** Pre-test-adjusted between-group comparisons (ANCOVA, estimated marginal means).

Variable	Time	Comparison	Adjusted diff.	95% CI	*p*	Hedges g
Nelson hand reaction test (ms)	Post-test	TGAG–ACG	−2.30	[−3.70, −0.90]	< 0.001	−0.27
		TGAG–CG	−3.71	[−5.09, −2.33]	< 0.001	−0.47
		ACG–CG	−1.41	[−2.79, −0.03]	0.042	−0.20
	Follow-up	TGAG–ACG	−3.53	[−5.08, −1.98]	< 0.001	−0.42
		TGAG–CG	−3.22	[−4.77, −1.67]	< 0.001	−0.42
		ACG–CG	0.30	[−1.22, 1.82]	1.000	0.04
Plate tapping test (s)	Post-test	TGAG–ACG	−0.07	[−0.19, 0.05]	0.415	−0.09
		TGAG–CG	−0.23	[−0.33, −0.13]	< 0.001	−0.27
		ACG–CG	−0.16	[−0.26, −0.06]	0.002	−0.19
	Follow-up	TGAG–ACG	−0.25	[−0.35, −0.15]	< 0.001	−0.32
		TGAG–CG	−0.22	[−0.32, −0.12]	< 0.001	−0.26
		ACG–CG	0.04	[−0.06, 0.14]	1.000	0.05
Modified bass dynamic balance test (points)	Post-test	TGAG–ACG	3.36	[1.91, 4.81]	< 0.001	1.08
		TGAG–CG	6.36	[4.93, 7.79]	< 0.001	1.78
		ACG–CG	3.01	[1.58, 4.44]	< 0.001	1.06
	Follow-up	TGAG–ACG	5.34	[3.67, 7.01]	< 0.001	1.43
		TGAG–CG	5.35	[3.68, 7.02]	< 0.001	1.39
		ACG–CG	0.01	[−1.64, 1.66]	1.000	~0.00
Pro-agility test (s)	Post-test	TGAG–ACG	−0.21	[−0.28, −0.14]	< 0.001	−0.79
		TGAG–CG	−0.27	[−0.34, −0.20]	< 0.001	−1.14
		ACG–CG	−0.06	[−0.13, 0.01]	0.081	−0.21
	Follow-up	TGAG–ACG	−0.28	[−0.38, −0.18]	< 0.001	−1.27
		TGAG–CG	−0.21	[−0.31, −0.11]	< 0.001	−0.84
		ACG–CG	0.07	[−0.03, 0.17]	0.240	~0.00

TGAG, target games activity group; ACG, active control group; CG, control group. Comparisons are based on pre-test-adjusted estimated marginal means from one-way ANCOVA models (covariate: pre-test score). Negative values indicate better performance for the first-listed group on Nelson, Plate Tapping, and Pro-Agility; positive values indicate better performance for the first-listed group on the Modified Bass test. Effect sizes (Hedges g) were calculated from pooled raw post-test/follow-up standard deviations rather than ANCOVA residual variance. 95% confidence intervals were computed using Bonferroni-adjusted critical values (df = 65) to maintain consistency with the Bonferroni-corrected p-values.

### Plate tapping test

The main effect of time was significant (*F*_(1.73, 114.48)_ = 19.83, *p* < 0.001, η*p*^2^ = 0.23), the main effect of group was not (*F*_(2, 66)_ = 0.30, *p* = 0.742, η*p*^2^ = 0.01), and the Time × Group interaction was significant (*F*_(3.47, 114.48)_ = 12.95, *p* < 0.001, η*p*^2^ = 0.28). The TGAG improved significantly from pre- to post-test (*p* < 0.001) and pre-test to follow-up (*p* < 0.001), with gains maintained through follow-up (*p* = 1.000). The ACG improved significantly from pre- to post-test (*p* < 0.001), but this improvement was significantly lost by follow-up (*p* < 0.001; pre-test–follow-up *p* = 1.000). No significant change occurred in the CG (all *p* = 1.000).

Pre-test-adjusted ANCOVA models (Table 6) showed a significant between-group difference at post-test (*F*_(2, 65)_ = 14.04, *p* < 0.001, η*p*^2^ = 0.30). Notably, the TGAG and ACG did not differ significantly from each other at post-test (adjusted difference = 0.07 s, *p* = 0.415), although both were significantly faster than the CG (TGAG: 0.23 s, *p* < 0.001; ACG: 0.16 s, *p* = 0.002). At follow-up, the between-group difference remained significant (*F*_(2, 65)_ = 22.03, *p* < 0.001, η*p*^2^ = 0.40), and the TGAG was now significantly faster than both the ACG (0.25 s, *p* < 0.001) and the CG (0.22 s, *p* < 0.001), while the ACG–CG difference was no longer significant (0.04 s, *p* = 1.000). This pattern indicates that the TGAG's advantage over the ACG on this measure emerged specifically during the retention period, as the ACG's post-test gains were lost by follow-up.

### Modified bass dynamic balance test

The main effects of time (*F*_(1.75, 115.60)_ = 64.17, *p* < 0.001, η*p*^2^ = 0.49) and group (*F*_(2, 66)_ = 11.89, *p* < 0.001, η*p*^2^ = 0.26), as well as the Time × Group interaction (*F*_(3.50, 115.60)_ = 39.94, *p* < 0.001, η*p*^2^ = 0.55), were all statistically significant. The TGAG showed a marked improvement from pre- to post-test (*p* < 0.001) and pre-test to follow-up (*p* < 0.001), which was maintained at follow-up (*p* = 0.583). In the ACG, the pre-to-post improvement was significant (*p* < 0.001), but a significant decline followed from post-test to follow-up (*p* < 0.001), with the pre-test–follow-up difference no longer significant (*p* = 1.000), indicating a loss of balance gains. No significant change occurred in the CG (all *p* = 1.000).

Pre-test-adjusted ANCOVA models (Table 6) confirmed significant between-group differences at both post-test (*F*_(2, 65)_ = 59.49, *p* < 0.001, η*p*^2^ = 0.65) and follow-up (*F*_(2, 65)_ = 40.74, *p* < 0.001, η*p*^2^ = 0.56). At post-test, all three pairwise comparisons were significant: the TGAG outperformed both the ACG (3.36 points, *p* < 0.001) and the CG (6.36 points, *p* < 0.001), and the ACG outperformed the CG (3.01 points, *p* < 0.001). At follow-up, the TGAG remained significantly superior to both the ACG (5.34 points, *p* < 0.001) and the CG (5.35 points, *p* < 0.001), while the ACG–CG difference was no longer significant (0.01 points, *p* = 1.000), confirming that the ACG had regressed to the CG level.

### Pro-agility test

The main effect of time (*F*_(1.69, 111.22)_ = 12.70, *p* < 0.001, η*p*^2^ = 0.16) and the Time × Group interaction (*F*_(3.37, 111.22)_ = 20.93, *p* < 0.001, η*p*^2^ = 0.39) were significant, whereas the main effect of group was not (*F*_(2, 66)_ = 2.21, *p* = 0.118, η*p*^2^ = 0.06). The TGAG improved significantly from pre- to post-test (*p* < 0.001) and pre-test to follow-up (*p* < 0.001), with gains maintained at follow-up (*p* = 1.000). No significant change was observed at any time point for the ACG (*p* values ranging from.054 to 1.000) or the CG (all *p* = 1.000).

In contrast to the raw between-group comparisons based on observed post-test means, pre-test-adjusted ANCOVA models (Table 6) revealed a significant between-group difference at post-test (*F*_(2, 65)_ = 53.48, *p* < 0.001, η*p*^2^ = 0.62): the TGAG had significantly faster change-of-direction speed than both the ACG (0.21 s, *p* < 0.001) and the CG (0.27 s, *p* < 0.001), while the ACG–CG difference approached but did not reach significance (0.06 s, *p* = 0.081). This between-group difference remained significant at follow-up (*F*_(2, 65)_ = 29.19, *p* < 0.001, η*p*^2^ = 0.47), with the TGAG continuing to outperform both the ACG (0.28 s, *p* < 0.001) and the CG (0.21 s, *p* < 0.001), while the ACG–CG difference remained non-significant (0.07 s, *p* = 0.240).

In summary, the Time × Group interaction was statistically significant for all four performance variables. The TGAG showed a significant improvement from pre- to post-test on every measure, and these gains were maintained across the 4-week follow-up period. The ACG showed a partial post-test improvement on the Nelson, Plate Tapping, and Modified Bass Dynamic Balance tests, but these gains were significantly lost by follow-up; no significant change was observed for the ACG on the Pro-Agility test. The CG showed no statistically significant change on any variable at any time point.

Pre-test-adjusted ANCOVA models provided a direct test of between-group differences in change (Table 6). Across all four outcomes, the TGAG differed significantly from both the ACG and the CG at follow-up, whereas the ACG no longer differed significantly from the CG at follow-up on any measure—indicating that the ACG's post-test advantage over the CG, where present, was not retained. At post-test, the pattern was more variable across outcomes: the TGAG was significantly superior to both control groups on the Modified Bass and Pro-Agility tests and on the Nelson test, but did not differ significantly from the ACG on the Plate Tapping test, where its advantage over the ACG emerged only at follow-up. Effect sizes (Hedges g) for the TGAG's advantage over the CG ranged from small (Nelson, Plate Tapping) to large (Modified Bass, Pro-Agility), indicating that the magnitude of the target-games effect differed across the outcome domains assessed.

## Discussion

The primary aim of this study was to examine the effect of a structured target games program on reaction time, upper-limb movement speed, dynamic balance, and change-of-direction speed in children aged 10–14 years, and to evaluate the retention of any resulting gains during a follow-up period. The principal finding was that the TGAG exhibited significant improvements across all four outcomes at post-test, and that these improvements were maintained—and, on three of the four outcomes, distinguished the TGAG from both control groups—at the 4-week follow-up. This pattern suggests that target-game-based interventions can produce gains that persist beyond the intervention period, rather than a purely transient effect, although longer-term follow-up is needed to establish true durability.

The coordination of visual perception with motor response is described as visual-motor integration (VMI; Kulp and Sortor, 2003), a process in which the sensory system relays visually perceived environmental information to the brain, which then determines and activates an appropriate motor response (Goodale, 1998). Interventions that target the integration of visual and motor domains jointly—rather than either domain in isolation—appear particularly effective in stimulating motor development (Pieters et al., 2012). This is consistent with reports that structured sports games in physical education positively affect adolescents' attention, reaction speed, spatial awareness, and motor coordination (Bashtovenko et al., 2021), and that sensory-motor training meaningfully improves visual perception and attentional skills, thereby supporting fine motor performance (Suri and Azadian, 2023). The target games used in the present intervention required a high degree of focus, aiming, and rapid motor decision-making; it is plausible that these demands engaged VMI-related processes, offering one possible mechanistic explanation for the performance gains observed, although no mechanistic variable was directly measured in this study.

The significant pre- to post-test improvement observed in the TGAG on the Pro-Agility Test (Table 5, *p* < 0.001), together with the significant between-group differences confirmed by the pre-test-adjusted ANCOVA at both post-test and follow-up (Table 6), is consistent with the premise that the target games intervention favorably influenced change-of-direction speed. Thieschäfer and Büsch (2022) reported that the relationship between perceptual-cognitive factors and motor performance in agility development strengthens with age, and that agility itself is trainable through appropriate intervention. Physical exercise has similarly been shown to benefit students' memory, attention, reaction speed and accuracy, and spatial awareness (Bashtovenko et al., 2021), while specific coordination training has been shown to produce greater gains in technical and coordinative ability than traditional methods (Ljach and Witkowski, 2010). The repeated decelerations, directional changes, and target-oriented positioning inherent to the target games may have contributed to the observed improvement in change-of-direction speed, although the specific neuromuscular or perceptual mechanisms underlying this change were not directly assessed in the present study. No significant change was observed in the CG, and although the ACG showed a significant post-test advantage over the CG on several other outcomes, no corresponding improvement was observed on this measure (Table 5). Taken together, these results indicate that the target games program was associated with improved change-of-direction speed relative to both control conditions, an effect that was sustained at the 4-week follow-up.

Examination of upper-extremity movement speed (Plate Tapping) and reaction time (Nelson Hand Reaction) showed that both the TGAG and the ACG exhibited statistically significant pre- to post-test improvement on both tests (Table 5), whereas the TGAG retained these gains at follow-up and the ACG's performance regressed toward baseline levels (Table 5). The pre-test-adjusted between-group comparisons (Table 6) indicated that this distinction was not uniform across the two outcomes at post-test: on the Nelson test, the TGAG already differed significantly from both control groups, whereas on the Plate Tapping test, the TGAG and ACG did not differ significantly from one another until follow-up, when the ACG's post-test gains had been lost. This pattern is broadly consistent with the literature: Henkaryansyah et al. (2024), in a 12-session process-focused experimental study in pétanque, reported statistically significant positive changes between first and second measurements, consistent with gains in shooting accuracy, focus, and coordination; Gohr Månsson et al. (2019) and Løhre et al. (2022) similarly found that structured target-shooting practice was associated with improved attention-related measures in school-aged children; and Szabo et al. (2021) reported that tasks requiring eye–hand coordination were associated with improved hand coordination and reaction speed in children. Kurniawati et al. (2026) similarly reported that a sensorimotor play program was associated with improved controlled hand-movement skills and increased focus. These findings are broadly consistent with the reaction- and hand-speed gains observed in the present study, although the Nelson Hand Reaction Test score in this study was based on the single best of three trials rather than a trial average (see Limitations), and the observed differences, while statistically significant, were modest in absolute magnitude (Hedges g = 0.20–0.47 for the TGAG's advantage over the control groups; Table 6).

Examination of dynamic balance scores showed a marked and statistically significant improvement from pre- to post-test in both the TGAG and the ACG (Table 5). At follow-up, the TGAG maintained its post-test balance scores, whereas the ACG lost its gains and regressed toward baseline (Table 5). The pre-test-adjusted between-group comparisons (Table 6) confirmed that this was the outcome with the largest and most consistent between-group separation in the study: at post-test, the TGAG, ACG, and CG all differed significantly from one another, and at follow-up the TGAG remained significantly superior to both control groups (Hedges *g* = 1.39–1.43) while the ACG had regressed to the CG level. This pattern of change in Modified Bass Balance scores is consistent with the possibility that the target games intervention engaged the proprioceptive system and neuromuscular control relevant to postural stability; proprioception is known to play a role in planning coordinated movement, maintaining balance, and managing body position (Westlake and Culham, 2007), although this mechanism was not directly assessed in the present study. Fitzgerald et al. (2010) reported that game-based exercise systems can be as effective as classical exercise in developing balance while sustaining high participant motivation; Terrazzo-Luna et al. (2024) reported that 8 weeks of structured recreational games were associated with substantial improvements in balance and coordination in young children; and Li and Xu (2026) similarly reported that rhythmic and coordinative movement interventions were associated with improved balance and gross motor skills in primary-school-aged children. The sudden dynamic postures and single-leg weight transfers demanded by the target games used in the present study may have contributed to the observed improvement in dynamic balance scores, a pattern broadly consistent with the findings of Terrazzo-Luna et al. (2024) and Li and Xu (2026); however, as with the other outcomes in this study, the specific physiological mechanism underlying this change was not measured directly and remains a plausible, rather than confirmed, explanation.

Another key finding of this study is that the TGAG's gains relative to both control groups were, on three of the four outcomes, still evident at the 4-week follow-up, and on the fourth (Plate Tapping), the TGAG's advantage over the ACG was present only at follow-up. Across all four outcomes, the ACG's post-test advantage over the CG, where present, was no longer significant by follow-up—a consistent pattern suggesting that the gains associated with the general recreational games program were comparatively short-lived, whereas those associated with the target games program were more persistent over the 4-week interval assessed. This is broadly consistent with reports that structured recreational exercise programs in children can produce effects that remain comparatively stable over a follow-up period (Altin, 2026), and with a wider body of literature suggesting that motor-skill gains achieved through planned play activities may show some durability beyond the intervention period (Henkaryansyah et al., 2024; Kurniawati et al., 2026; Li and Xu, 2026; Terrazzo-Luna et al., 2024; Van Beurden et al., 2002). However, retention was assessed at a single time point 4 weeks after the intervention, and whether this pattern extends to longer intervals remains to be determined; the mechanisms through which regular, structured play might support the persistence of acquired motor skills were not directly examined in the present study.

### Limitations

Several methodological limitations of this study warrant consideration when interpreting the findings.

First, only male participants were included, based on the rationale that sex-based differences in motor development are pronounced in the 10–14 age range; consequently, the findings cannot be generalized to female adolescents, and future studies should examine whether comparable effects are observed in girls or in mixed-sex samples.

Second, the sample was drawn from a single city (Konya) and a single school, with a modest number of participants per group (*n* = 22–24). Although the single-school design was a deliberate methodological choice to standardize implementation conditions, it nonetheless limits generalizability to other schools or cultural contexts.

Third, outcome assessors were not formally blinded to group allocation, and formal inter-rater or test–retest reliability statistics for the four motor tests were not computed. Although all measurements were independently cross-checked twice by a separate team not involved in the intervention, this does not fully substitute for blinded assessment, and the possibility of measurement bias—particularly for tests involving an element of subjective judgment, such as balance scoring—cannot be entirely ruled out. Similarly, because sequence generation and allocation were both carried out by the lead researcher rather than an independent party, the possibility of allocation-related bias, although mitigated by the stratified and computer-based randomization procedure, cannot be entirely excluded. In addition, the Nelson Hand Reaction Test score was based on the single best of three trials rather than an average, which is a common field-testing convention but may inflate estimates of change if trial variability differed across groups or measurement occasions; this cannot be fully evaluated as individual trial data were not retained for later analysis. A mild deviation from normality was also observed for the CG's follow-up Modified Bass Dynamic Balance scores (Shapiro–Wilk *p* = 0.037); although the ANCOVA model for this outcome is generally robust to minor departures from normality given the sample size, this should be considered when interpreting the follow-up comparisons for this measure.

Fourth, exercise intensity was not objectively monitored in either intervention arm; heart rate, respiratory rate, and rating of perceived exertion were not recorded, and both programs were delivered at a self-selected, recreational pace with no maximal-effort demand. Although session duration, structure, and reward scheme were matched between the TGAG and the ACG, the comparison between arms should therefore be interpreted at the level of session content and structure rather than verified physiological load. Relatedly, contamination between arms and incomplete reporting of control-condition content are recognized risks in active-control designs (Ehlers et al., 2016; Byrd-Bredbenner et al., 2017); the present single-school, individually randomized design cannot fully exclude either, although the content of both arms is described in the Methods and Table 1, and full session-by-session protocols are available from the author upon request. To support transparency on this point. In addition, the recreational target games program was not subsequently offered to the ACG or CG after study completion; a wait-list design, in which control participants receive the intervention after the follow-up period, would address this equity consideration in future implementations.

Fifth, retention of the intervention's effects was assessed at a single follow-up point 4 weeks after the intervention; whether the observed gains persist over a longer time frame (e.g., 3 to 12 months) remains unknown.

Finally, participants' daily dietary habits, physical activity outside the intervention, and sleep quality and duration could not be directly controlled during the study period. As noted by Terrazzo-Luna et al. (2024), nutrition, fatigue, and sleep-related variables can particularly influence children's reaction time and motor performance.

## Conclusion

This study found that a structured target games program produced statistically significant improvements in change-of-direction speed, upper-limb movement speed, reaction time, and dynamic balance in boys aged 10–14 years, and that these gains distinguished the TGAG from both an active and a passive control group on most outcomes at a 4-week follow-up. The post-test improvements observed in the Active Control Group were, across all four outcomes, not retained at follow-up, suggesting that the gains associated with the general recreational games program used in this study were comparatively short-lived under the conditions tested. Effect sizes for the TGAG's advantage over the control groups varied considerably across outcomes, from small for reaction time and upper-limb movement speed to large for dynamic balance and change-of-direction speed, indicating that the practical significance of the intervention may differ by outcome domain. These findings should be interpreted in light of the study's limitations, including the single-school design, the absence of assessor blinding, and the lack of an objective measure of exercise intensity in either intervention arm. Within these constraints, target-oriented recreational games appear to be a promising, low-cost activity that schools could consider as part of structured physical activity programming; larger, preregistered trials that include female participants, longer follow-up periods, and objective intensity monitoring are needed before broader recommendations can be made.

## Data Availability

The raw data supporting the conclusions of this article will be made available by the authors, without undue reservation.
